# H_2_S biotreatment with sulfide-oxidizing heterotrophic bacteria

**DOI:** 10.1007/s10532-018-9849-6

**Published:** 2018-08-23

**Authors:** Ningke Hou, Yongzhen Xia, Xia Wang, Huaiwei Liu, Honglei Liu, Luying Xun

**Affiliations:** 1 State Key Laboratory of Microbial Technology, Shandong University, Jinan, 250100 People’s Republic of China; 2 School of Molecular Biosciences, Washington State University, Pullman, WA 99164-7520 USA

**Keywords:** Sulfide oxidation, Heterotrophic bacteria, Immobilized cells, Sulfide:quinone oxidoreductase

## Abstract

Many industrial activities produce H_2_S, which is toxic at high levels and odorous at even very low levels. Chemolithotrophic sulfur-oxidizing bacteria are often used in its remediation. Recently, we have reported that many heterotrophic bacteria can use sulfide:quinone oxidoreductase and persulfide dioxygenase to oxidize H_2_S to thiosulfate and sulfite. These bacteria may also potentially be used in H_2_S biotreatment. Here we report how various heterotrophic bacteria with these enzymes were cultured with organic compounds and the cells were able to rapidly oxidize H_2_S to zero-valence sulfur and thiosulfate, causing no apparent acidification. Some also converted the produced thiosulfate to tetrathionate. The rates of sulfide oxidation by some of the tested bacteria in suspension, ranging from 8 to 50 µmol min^−1^ g^−1^ of cell dry weight at pH 7.4, sufficient for H_2_S biotreatment. The immobilized bacteria removed H_2_S as efficiently as the bacteria in suspension, and the inclusion of Fe_3_O_4_ nanoparticles during immobilization resulted in increased efficiency for sulfide removal, in part due to chemical oxidation H_2_S by Fe_3_O_4_. Thus, heterotrophic bacteria may be used for H_2_S biotreatment under aerobic conditions.

**Electronic supplementary material:**

The online version of this article (10.1007/s10532-018-9849-6) contains supplementary material, which is available to authorized users.

## Introduction

H_2_S is produced by various industrial and natural activities, such as petroleum refining, methane-containing biogas production, wastewater treatment, and food processing (Eikum and Storhaug 1986; Hughes et al. 2009; Janssen et al. 2009). H_2_S is malodorous at low levels and toxic at high levels, inhibiting aerobic respiration to humans and microorganisms (Nicholls and Kim 1982). It can also be problematic in sewer systems, causing corrosion (Zhang et al. 2008). Under neutral pH, H_2_S and HS^−^ are the major species of sulfide, as pKa1 is 6.9 and pKa2 is > 12 (Kabil and Banerjee 2010).

Biofiltration is often used in H_2_S removal from waste gas or waste water because of its effectiveness, low energy consumption and minimal by-product generation compared to chemical and physical treatments (Sorokin 1994). Researchers have tried different reactor designs, various packing materials and nutrients for biofiltration to achieve stable efficiency of H_2_S elimination (Ben Jaber et al. 2016a, b; Gerrity et al. 2016; Li et al. 2008). However, biofiltration is limited to the use of a few of chemolithotrophic sulfur-oxidizing bacteria, such as *Thiobacillus* spp., *Acidithiobacillus* spp. and green sulfur bacterium (Pokorna and Zabranska 2015). A problem of using these sulfur-oxidizing bacteria is the production of sulfuric acid, leading to acidification of the liquid phase; however, the problem can be prevented if oxygen supply is restricted (Dolejs et al. 2015; Janssen et al. 1998; Sorokin et al. 2008; Wang et al. 2016). When acidification occurs, alkaline materials are added to neutralize the acidified liquid, which increases the cost (Gerrity et al. 2016; Mora et al. 2015; Pokorna and Zabranska 2015). Although some sulfide oxidizing bacteria such as *Acidithiobacillus* spp. can tolerate acidic conditions (Ben Jaber et al. 2016b), the acid effluent is also a source of pollution. Further, acidification does not favor H_2_S absorption for microbial consumption (Hughes et al. 2009). Consequently, a large reactor volume is required to increase the retention time of waste gas for efficient removal of H_2_S.

Although there were sporadic reports on utilizing heterotrophic bacteria for H_2_S removal under aerobic conditions, the genes and enzymes involved in the process were unknown (Chung et al. 1996). Recently, heterotrophic bacteria have been discovered to oxidize sulfide with sulfide:quinone oxidoreductase (SQR) and persulfide dioxygenase (PDO) (Luebke et al. 2014; Xin et al. 2016). Bacteria with *sqr* and *pdo* genes are able to oxidize sulfide produced from sulfur-containing organic compounds, such as cysteine. When the genes are deleted, the mutant cannot oxidize the self-produced sulfide and release H_2_S to the gas phase (Xia et al. 2017). Many common soil bacteria, such as *Bacillus* spp. and *Pseudomonas* spp. possess *sqr* and *pdo* genes, and their efficiency to oxidize sulfide at low levels has been demonstrated (Xia et al. 2017). However, it is unclear whether all heterotrophic bacteria with *sqr* and *pdo* genes can rapidly oxidize sulfide at high levels and what they oxidize sulfide to. Thus, here we want to make a thorough investigation on the possibility of these bacteria for applications in H_2_S biotreatment. Here, we investigated whether heterotrophic bacteria could effectively oxidize exogenous H_2_S.

## Materials and methods

### Bacterial strains and culture condition

The bacterial strains used in this study are listed in Table 1. Lysogeny broth (LB) medium was used for culturing most bacteria, and D-sorbitol medium (DM) was used for *Gluconobacter oxydans* 621H (Yang et al. 2008). *Zunongwangia profunda* SM-A87 was cultivated in a medium composed of 10 g L^−1^ peptone, 5 g L^−1^ yeast extract and artificial sea water (Qin et al. 2007). Most of the bacteria were incubated at 30 °C, and *E. coli* BL21 was incubated at 37 °C.

**Table 1:** The rates of sulfide oxidation by tested and reported bacteria

Strains	Sulfide oxidation rate^a^	Reaction condition
*T. thiooxidans*	158^b^	Aerobic
*T. denitrificans*	80^c^	Anaerobic and nitrate
*A. thioparus* TK-m	> 59^d^	Aerobic
*G. oxydans* 621H	50.1 ± 6.7^e^	Aerobic
*T. ferrooxidans*	48^b^	Aerobic
*Chlorobium*	12^f^	Light
*P. aeruginosa* PAO1	9.5 ± 0.5^e^	Aerobic
*P. putida* S16	8.4 ± 0.5^e^	Aerobic
*B. cepacia* ATCC 25416	5.6 ± 1.1^e^	Aerobic
*S. marcescens* ATCC 13880	1.5 ± 0.2^e^	Aerobic
*Z. profunda* SM-A87	0.25 ± 0.04^e^	Aerobic
*B. cereus* ATCC 10876	0.19 ± 0.02^e^	Aerobic
*S. aureus* ATCC 6538P	0.08 ± 0.01^e^	Aerobic

^a^The tested bacteria are heterotrophs with SQR and PDO, and the reported bacteria are chemolithotrophs or phototroph. The unit of sulfide oxidation rate is µmol min^−1^ g^−1^ of cell dry weight

^b^Oprime et al. (2001)

^c^Sublette and Sylvester (1987)

^d^Kanagawa and Mikami (1989)

^e^Our data

^f^Kim and Chang (1991)

### Sulfide oxidation analysis

Bacteria were cultivated for about 24 h when the bacteria reached to stationary phase and OD_*600nm*_ was greater than 4. The cells were harvested by centrifugation (5000×*g*, 10 min) and resuspended to a turbidity of 2 at 600 nm in 50 mM HEPES buffer, pH 7.4 containing 50 μM diethylenetriaminepentaacetic acid (DTPA) to minimize spontaneous sulfide oxidation (Hughes et al. 2009). 10 mL of the cell suspension was transferred to a 50-mL centrifuge tube. Freshly prepared NaHS (Sigma; CAS: 207683-19-0) solution was added to initiate the reaction. The tube was capped tightly and incubated at 30 °C with shaking of 180 rpm. Sulfide was analyzed at various time intervals by using a diamine reagent (Fogo and Popowsky 1949). After the reaction, the cells were harvested again, washed with deionized water and lyophilized for 12 h to obtain cell dry weights (Xia et al. 2012). Sulfide oxidation rate (*q*) was determined by the following equation:

$$q = \frac{{\left( {C_{0} - C_{t} } \right) \times V}}{m \times T}$$

where *C*_0_ and *C*_*t*_ were the sulfide concentrations (µM) at the beginning and the time of sampling; *V* was the volume of solution (mL), *m* was the dry weight (g) of cells, and *T* was the incubation time (min). Sulfide oxidation analysis with immobilized cells was similar in 50 mM HEPES buffer, pH 7.4.

The sulfide oxidation experiments with multiple sulfide additions were done in a mineral medium (MM). One liter of MM contained 0.3 g of (NH_4_)_2_SO_4_, 1.5 g of Na_2_HPO_4_, 0.5 g of KH_2_PO_4_, 0.095 g of MgCl_2_, 0.033 g of CaCl_2_, 5 g of glucose, 0.1 g of pantothenic acid (Gupta et al. 2001) and 1 mL of a trace mineral solution. The trace mineral solution contained 4.5 g L^−1^ FeSO_4_·7H_2_O, 1.44 g L^−1^ ZnSO_4_·7H_2_O, 0.86 g L^−1^ MnSO_4_·H_2_O, 0.16 g L^−1^ CuSO_4_, 0.28 g L^−1^ CoSO_4_·7H_2_O, 0.06 g L^−1^ H_3_BO_3_ and 10 mL L^−1^ H_2_SO_4_ (conc.). The suspended or immobilized cells were in 50-mL centrifuge tubes containing 10 mL of MM and incubated at 30 °C, 180 rpm. Sulfide was added to 2 mM to initiate the reaction in each cycle. After each reaction, the immobilized cells were washed with water and re-incubated in fresh MM at 30 °C, 180 rpm. Each reaction cycle was carried out in every 12 h.

### End-products analysis

Bacteria were harvested when they were cultivated to stationary phase and resuspended in 50 mM HEPES buffer to a turbidity of 8 at 600 nm. Ten mL of the cell suspension was transferred to a 50-mL centrifuge tube. Freshly prepared sodium sulfide was added to initiate the reaction. The tube was capped tightly and incubated at 30 °C, 180 rpm. The products and sulfide were analyzed at various time intervals.

To test the changes in pH after sulfide oxidation by magnetically immobilized cells, the experiments were done in the 50-mL centrifuge tube containing 10 mL of unbuffered 0.9% NaCl (pH 7) at 30 °C, 180 rpm. Sulfide was added to 2 mM to initiate the reaction in each cycle, and the next cycle started when sulfide was completely oxidized. The pH was tested at the beginning and the end of each cycle. The products were detected at the end of each cycle.

Cellular zero-valence sulfur, including polysulfide, persulfide, and elemental sulfur, was detected by the cyanolysis method (Xin et al. 2016). Sulfate, thiosulfate and sulfite were detected by using ion chromatography (ICS-1100 system; Dionex) with a mobile phase of 20 mM KOH at a flow rate 1 mL per min. The retention times of sulfite, sulfate and thiosulfate were 7.4 min, 7.9 min and 25.7 min, respectively (Liu et al. 2014). For tetrathionate detection, ion chromatography (ICS-1100 system; Dionex) was used with a moblie phase of 15 mM KOH at a flow rate 0.9 mL per min.

### Preparation of Fe_3_O_4_ nanoparticles

The Fe_3_O_4_ nanoparticles were prepared according to a reported method with some modifications (Wang et al. 2007). 58.75 g of FeCl_3_·6H_2_O and 21.5 g of FeCl_2_·4H_2_O were dissolved in 1.5 L of distilled water at 30 °C with a gentle stream of N_2_ bubbling to minimize the oxidation of Fe^2+^. NH_3_ (8 M) was then slowly injected into the mixture with vigorous stirring until the pH reached 10. After precipitation, the Fe_3_O_4_ particles were repeatedly washed with distilled water until the pH became constant and they were lyophilized for 24 h to remove water. The lyophilized product was pulverized in an agate mortar to form Fe_3_O_4_ powder. 1.5 g of Fe_3_O_4_ powder was added into 10 mL of distilled water and dispersed by ultrasonic disruption (20 kHz; 10 min; Q125; QSonica) to form a stable suspension.

### Preparation of nonmagnetically immobilized and magnetically immobilized cells

The nonmagnetically and magnetically immobilized cells were prepared according to a reported method (Wang et al. 2007) with some modifications. Briefly, bacteria were harvested when they were cultivated to stationary phase and resuspended in a small volume of distilled water. The alginate gel (3% [wt/vol]) and cell suspension were mixed at a ratio of cell wet weight to dry alginate powder of 3 (wt/wt). Nonmagnetically immobilized cells were formed by extruding the mixture through a syringe into 0.2 M CaCl_2_ and incubated for 2 h to let the beads harden. For preparing magnetically immobilized cells, an 80 µL/mL Fe_3_O_4_ particle suspension was added to the above-mentioned mixture of alginate and cell suspension, and the procedure was the same as above. Nonmagnetically immobilized inactive cells and magnetically immobilized inactive cells were prepared in the same way by using heat-inactivated (boiling water 30 min) cells.

### Scanning electron microscope (SEM)

The alginic gel beads were first fixed with 2.5% of glutaraldehyde in 0.2 M phosphate buffer (pH 7.0) for 12 h, washed three times in the 0.2 M phosphate buffer (pH 7.0). The treated beads was further fixed with 1% of OsO_4_ for 2 h and washed three times in the 0.2 M phosphate buffer. The specimen was dehydrated by using a series of ethanol (30%, 50%, 70%, 80%, 90%, 95% and 100%) for 15 to 20 min at each soaking. In the end, the specimen was dehydrated in Alpha 1–2 LD plus freeze dryer (CHRIST, Germany) for 75 min, coated with gold, and observed under SEM by using a QUANTA FEG 250 Scanning Microscope (FEI company, USA).

### Analysis of TsdA in sequenced bacterial genomes and construction of SQRs and PDOs phylogenetic trees

A microbial genomic protein sequence set from NCBI updated until November 11, 2017 was downloaded for thiosulfate dehydrogenase (TsdA) search. The query sequences of TsdA were reported TsdA(Denkmann et al. 2012; Kurth et al. 2016) and were used to search the database by using Standalone BLASTP algorithm with conventional criteria (e-value ≤ 1 e^−10^, coveryage ≥ 40%, identity ≥ 30%) to obtain TsdA candidates from 8286 bacterial genomes. A conserved domain COG3258 of TsdAs was used as standard feature for further filtration of TsdA candidates. The selected candidates were then manually screened by reported conserved amino acid sequences (Denkmann et al. 2012). The candidates were combined with the query TsdAs for phylogenetic tree analysis by using ClustalW for alignment and MEGA version 7.0 program for neighbor-joining tree building with a pairwise deletion, *p*-distance distribution, and bootstrap analysis of 1000 repeats as parameters(Kumar et al. 2016). The candidates that were in the same clade with the query TsdAs were identified as putative TsdA. The phylogenetic trees of 11 SQRs and 9 PDO (Table S1) were constructed by using a neighbor-joining analysis with the MEGA version 7.0 program, running a pairwise deletion, *p*-distance distribution, and bootstrap analysis of 1000 repeats.

## Results

### Heterotrophic bacteria with *sqr* and *pdo* oxidize exogenous sulfide

**Fig. 1:**
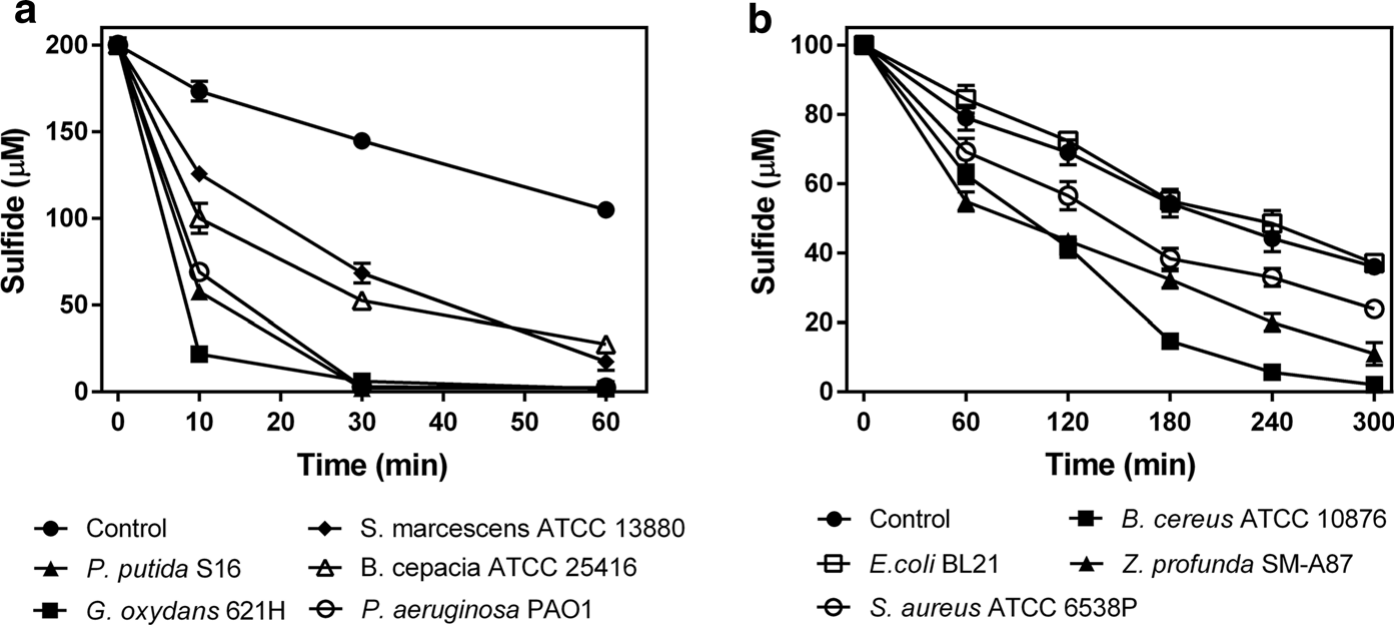
Heterotrophic bacteria containing *sqr* and *pdo* oxidized exogenous sulfide. Cells were cultured in LB, harvested, wash and re-suspended at OD_600nm_ of 2 in 50 mM HEPES buffer, pH 7.4, containing 50 µM DPTA. NaHS was added to 100 or 200 µM to initiate the reaction. **a** Six bacteria oxidized exogenous sulfide rapidly. **b** Three bacteria slowly oxidized exogenous sulfide; *E. coli* did not. Control contained no cells. Averages (n ≥ 3) with standard deviations (error bar) were shown

We selected eight heterotrophic bacteria containing *sqr* and *pdo* (Xia et al. 2017), grew them in a rich medium, harvested cells, and tested the resting cells for the oxidation of exogenous sulfide. They all oxidized sulfide, but the control *E. coli* BL21(DE3) without *sqr* and *pdo* did not (Fig. 1a, b). Most sulfide-oxidizers had higher activities of sulfide oxidation at the stationary phase of growth than at the log phase, but *Gluconobacter oxydans* 621H had similar rates at all phases of growth. Thus, all the cells were cultured to the stationary phase, harvested, and used for sulfide oxidation. The results showed all tested heterotrophic bacteria with *sqr* and *pdo* oxidized sulfide. *Gluconobacter oxydans* 621H, *P. putida* S16, *P. aeruginosa* PAO1 and *B. cepacia* ATCC 25416 were more efficient for sulfide oxidation (Fig. 1a) than others (Fig. 1b). The slow decrease of sulfide in the control was attributed to volatilization and abiotic oxidation of sulfide (Xin et al. 2016). The sulfide oxidation rates of these heterotrophic bacteria with *sqr* and *pdo* ranged from 0.1 to 50 µmol min^−1^ g^−1^ of cell dry weight, showing that some of the tested heterotrophic bacteria had rates of sulfide removal sufficient for H_2_S biotreatment, as the unit rates are not too far behind those reported rates of chemolithotrophic bacteria (Table 1).

**Fig. 2:**
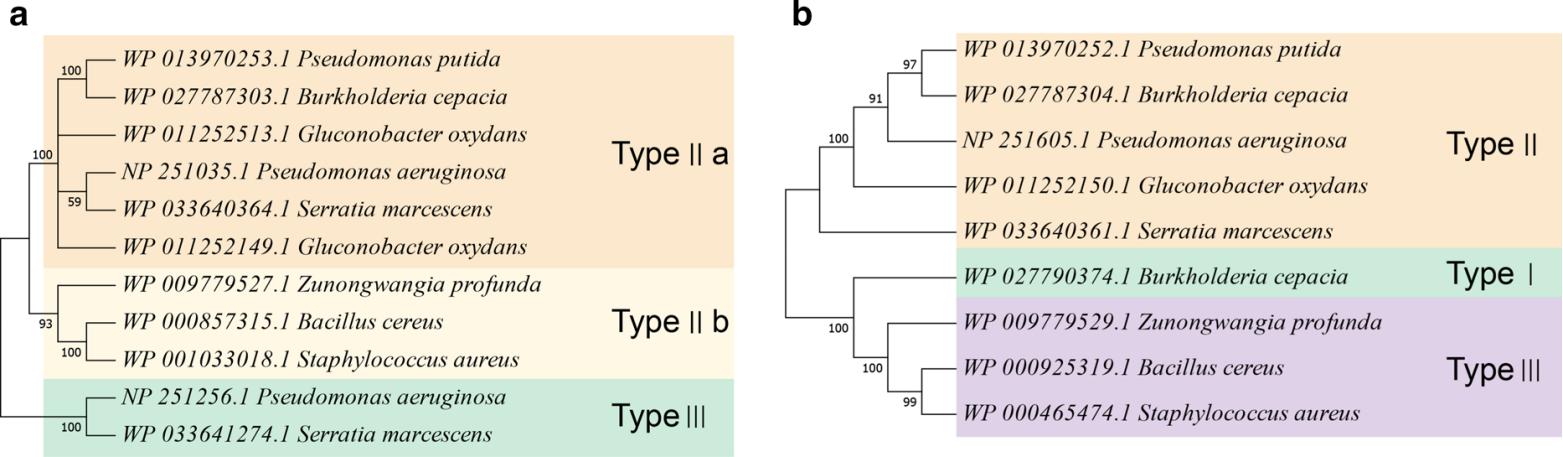
The phylogenetic trees of SQRs and PDOs from the eight heterotrophic bacteria. 11 SQRs (**a**) and 9 PDOs (**b**) were used for phylogenetic tree construction with reference sequences

SQRs are characterized into six types, and the PDOs are classified into three (Gregersen et al. 2011; Xia et al. 2017). To evaluate whether the types of SQRs and PDOs may affect the degradation rate, we constructed phylogenetic trees with the 11 SQRs and nine PDOs from the eight tested bacteria. The SQRs were grouped into type II and type III, and the type II SQRs were further divided into types IIa and types IIb. The fast sulfide-oxidizing bacteria *G. oxydans* 621H, *P. putida* S16, *P. aeruginosa* PAO1, and *B. cepacia* ATCC 25416 all contained types IIa SQRs. The PDOs were mapped into all three types, and the fast sulfide-oxidizing bacteria all contained the type II PDOs (Fig. 2b). Interestingly, *G. oxydans* 621H had two *sqr* genes, encoding two type IIa SQRs, and one of them was adjacently linked to a *pdo* gene on genome. The *sqr* and *pdo* genes of *P. putida* S16 and *B. cepacia* ATCC 25416 were also adjacently linked on genome (Fig. 2a, b). However, the slow sulfide-oxidizing bacterium *S. marcescens* ATCC 13880 also harbored type IIa SQR and type II PDO (Fig. 2a, b). The results suggest that bacteria with type IIa SQRs and type II PDOs are likely fast sulfide oxidizers, but they need to be verified.

### The products of H_2_S oxidation

**Fig. 3:**
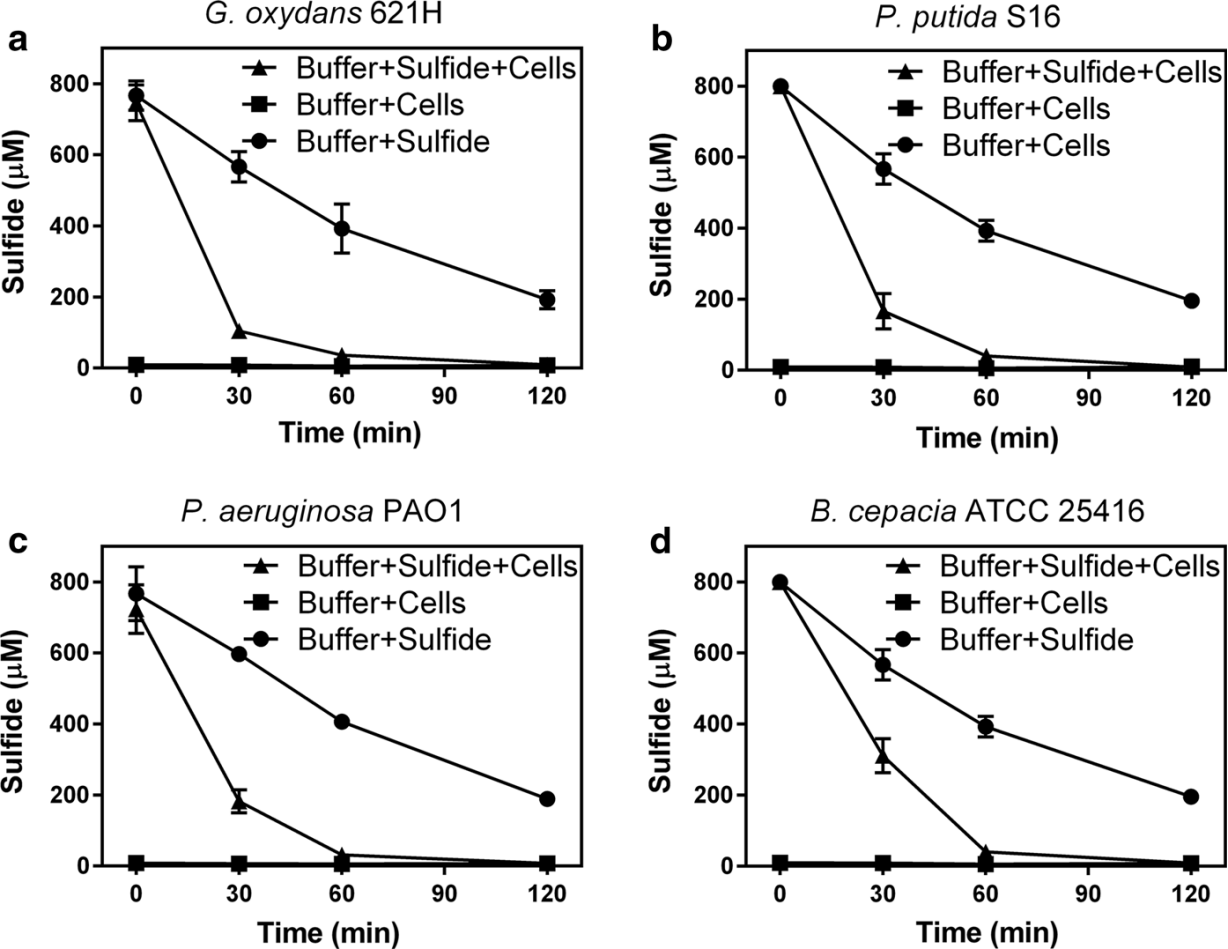
H_2_S oxidation by heterotrophic bacteria. Cells were harvested in LB, wash and re-suspended in 50 mM HEPES buffer (pH 7.4) at OD_600_nm of 8. NaHS was added to 750 ± 50 µM to initiate the reaction. **a** *G. oxydans* 621H; **b** *P. putida* S16; **c** *P. aeruginosa* PAO1; **d** *B. cepacia* ATCC 25416; sulfide only (closed square); cells only (closed square); cells and sulfide (closed triangle). All data are average of at least three samples with standard deviation (error bar)

**Fig. 4:**
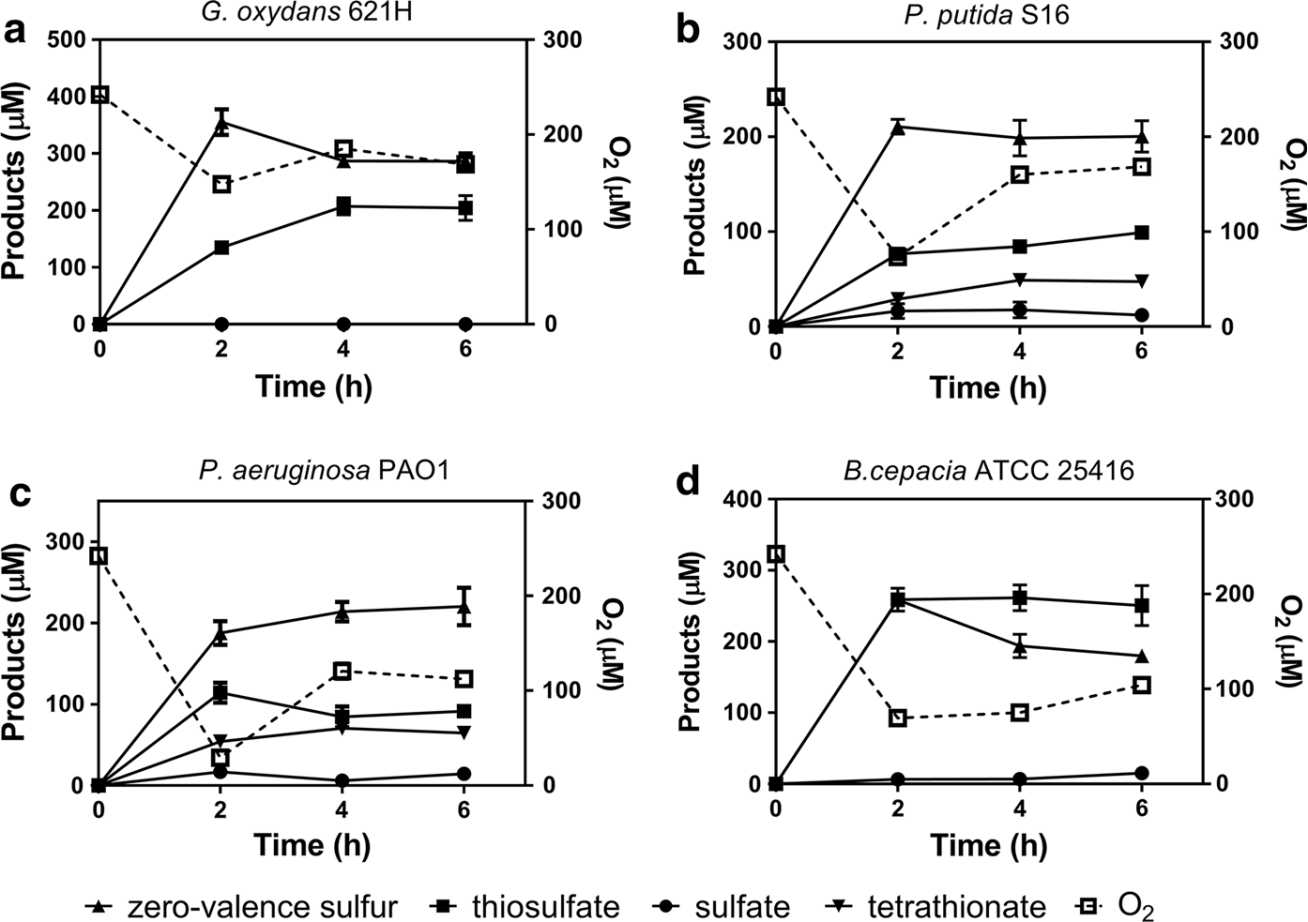
The products of H_2_S oxidation by selected bacteria. The conditions were the same as described in Fig. 1 legend. Sulfide was completely oxidized within 2 h and products were analyzed. **a** *G. oxydans* 621H; **b** *P. putida* S16; **c** *P. aeruginosa* PAO1; **d** *B. cepacia* ATCC 25416. Averages (n ≥ 3) with standard deviations (error bar) were shown

Four bacteria that rapidly oxidized sulfide (Fig. 1a) were used to analyze the end products from sulfide oxidation. To facilitate the detection of end products, sulfide was added to resuspended cells to a final concentration of 750 ± 50 µM, which was completely oxidized within 2 h (Fig. 3). The products were then detected. Sulfite was not detectable in all the samples. Thiosulfate and zero-valence sulfur were the main products of *G. oxydans* 621H and *B. cepacia* ATCC 25,416 (Fig. 4). *Pseudomonas putida* S16, *P. aeruginosa* PAO1 also produced tetrathionate (Fig. 4). Within the first 2 h of the reaction, sulfide was completely consumed by the tested bacteria, and the oxygen in the reaction mixture was decreased from about 240 μM to different levels, ranging from 25 μM with *P. aeruginosa* PAO1 to 150 μM with *G. oxydans* 621H (Fig. 4). After the first sampling at 2 h of incubation, further reduction in oxygen content was not observed. The increase in dissolved oxygen was likely due to sampling, during which the tube was opened. The product composition did not change much after the first 2-h incubation. *Gluconobacter oxydans* 621H that consumed the least amount of oxygen accumulated the most zero-valence sulfur (Fig. 4a). The two *Pseudomonas* spp. that consumed the most oxygen in the first two h also produced tetrathionate (Fig. 4b, c).

### Bacteria with TsdA can convert thiosulfate to tetrathionate

The production of tetrathionate is likely due to the presence of TsdA in *P. putida* S16 and *P. aeruginosa* PAO1. *Pseudomonas* spp. are known to use thiosulfate dehydrogenase (TsdA) to oxidize thiosulfate to tetrathionate, which may provide some supplemental energy (Denkmann et al. 2012; Sorokin et al. 1999). Thus, the distribution of TsdA were analyzed among 8286 microbial genomic protein sequences (NCBI updated until November 11, 2017) by using BLAST search, and then confirmed with the conserved domain and conserved amino acid sequence and phylogenetic tree analysis (Denkmann et al. 2012). 1275 identified TsdA distributed in 1112 bacterial genomes, including 553 Betaproteobacteria, 294 Gammaproteobacteria, 115 Alphaproteobacteria, 91 Epsilonproteobacteria, 11 Bacilli, 9 Synechococcales, 8 Flavobateria, 7 Sphingobacteria, 6 Deltaproteobacteria, and other classes with a few genomes containing TsdA. Of the Gammaproteobacteria, there are 178 *Pseudomonas* genomes contained TsdA species. Thus, when bacteria with TsdA are used for biotreatment, tetrathionate may also be produced.

### Cell growth and sulfide oxidation are coupled

*Pseudomonas putida* S16 consumed 1 g of glucose and 0.29 g of (NH_4_)_2_SO_4_ to yield 0.24 g of biomass as our test in MM (Eq. 1). Then, the harvested *P. putida* S16 cells (0.24 g dry weight) oxidized 726 μmol of H_2_S to about 242 μmol of S^0^, 121 μmol of H_2_S_2_O_3_, 61 μmol of H_2_S_4_O_3_ in a buffer in 6 h (Fig. 4) (Eq. 2). Here the bacterial cells were for catalysis, and no carbon source was provided to support growth.

1 $$2 {\text{C}}_{ 6} {\text{H}}_{ 1 2} {\text{O}}_{ 6} + {\text{NH}}_{ 3} + 7 {\text{O}}_{ 2} \to {\text{C}}_{ 5} {\text{H}}_{ 7} {\text{O}}_{ 2} {\text{N}} + 7 {\text{CO}}_{ 2} + 10{\text{H}}_{ 2} {\text{O}}$$

2 $$1 2 {\text{H}}_{ 2} {\text{S}} + 9 {\text{O}}_{ 2} \to 4 {\text{S}}^{0} + 2 {\text{H}}_{ 2} {\text{S}}_{ 2} {\text{O}}_{ 3} + {\text{H}}_{ 2} {\text{S}}_{ 4} {\text{O}}_{ 3} + 9 {\text{H}}_{ 2} {\text{O}}$$

### Sulfide oxidation by immobilized cells and free cells

**Fig. 5:**
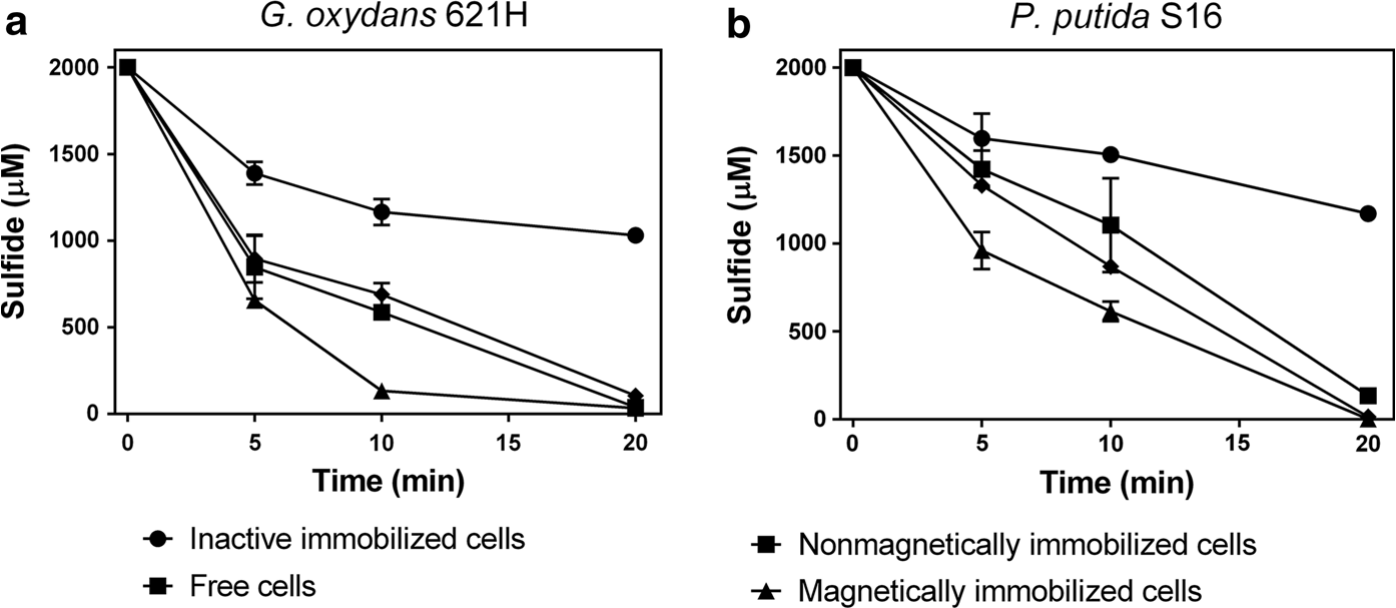
Sulfide oxidation by free cells, immobilized cells, and magnetically immobilized cells. The experiments were carried out in the 50-mL centrifuge tube containing 10 mL of 50 mM HEPES buffer (pH 7.4) at 30 °C, 180 rpm. The biomass was equivalent between free cells (OD_600_ of 8) and immobilized cells. NaHS was added to 2 mM to initiate the reaction. **a** *G. oxydans* 621H immobilized cells and free cells; **b** *P. putida* S16 immobilized cells and free cells. Averages (n ≥ 3) with standard deviations (error bar) were shown

We immobilized *G. oxydans* 621H and *P. putida* S16 into alginate gel beads as they showed the high rates of sulfide oxidation (Fig. 1, Table 1), and the immobilized cells had equivalent sulfide-oxidation rates to those of the suspended cells (Fig. 5). When cells were coimmobolized with the Fe_3_O_4_ nanoparticles into gel beads, the activity of sulfide oxidation increased by about 30% (Fig. 5). Further research found that Fe_3_O_4_ nanoparticles-containing beads without cells also catalyzed sulfide oxidation, consuming 400 μM more sulfide than the control with the beads containing no Fe_3_O_4_ in 40 min (Fig. S3), suggesting the ferric iron in Fe_3_O_4_ nanoparticles contributes to the increased sulfide oxidation rate.

**Fig. 6:**
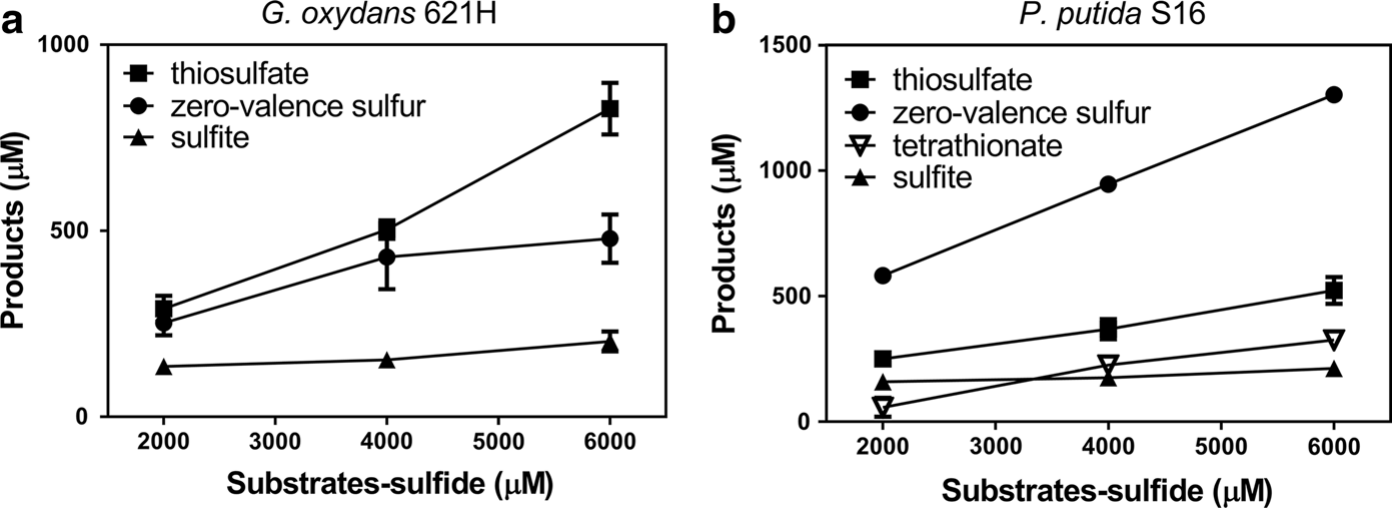
The products of magnetically immobilized cells. The experiments were carried out in the 50-mL centrifuge tube containing 10 mL of 0.9% NaCl at 30 °C on a reciprocal shaker at 200 rpm. NaHS was added to 2 mM to initiate the reaction in each cycle and the products were detected at the end of each cycle. **a** *G. oxydans* 621H magnetically immobilized cells; **b** *P. putida* S16 magnetically immobilized cells. Averages (n ≥ 3) with standard deviations (error bar) were shown

The sulfide oxidation products by immobilized cells of *G. oxydans* 621H and *P. putida* S16 containing Fe_3_O_4_ nanoparticles were also mainly zero-valence sulfur and thiosulfate (Fig. 6). The total amount of the detectable sulfur was less than half of the added sulfide, and the rest might be trapped inside the gel beads, which could not be detectable without releasing from the beads. Different from the products of free cells (Fig. 4), there was also a small amount of sulfite in the products of immobilized cells with Fe_3_O_4_ nanoparticles. Further research found that the same amount of Fe_3_O_4_ powder without cells reacted with sulfide under aerobic conditions to produce about 20% sulfite, 60% thiosulfate and 20% zero-valence sulfur, suggesting that Fe_3_O_4_ affected the product composition. However, the reaction rate with Fe_3_O_4_ was slower, at about 25% of that by immobilized *G. oxydans* 621H with Fe_3_O_4_ nanoparticles.

### No acidification after sulfide oxidation

Acidification during sulfide biotreatment is a potential problem when sulfuric acid is produced by chemolithotrophs with sufficient supply of O_2_ (Pokorna and Zabranska 2015). When immobilized *G. oxydans* 621H and *P. putida* S16 with Fe_3_O_4_ nanoparticles were tested, the pH in the liquid phase (0.9% NaCl solution) was slightly increased after three cycles of sulfide oxidation, consuming total 6 mM NaHS (Table 2). The lack of acidification is likely due to the production of zero-valence sulfur, thiosulfate, and tetrathionate instead of sulfate (Fig. 6).

**Table 2:** The change of pH after sulfide oxidation

Strains	pH			
	Initiation	First cycle	Second cycle	Third cycle
*G. oxydans* 621H	7.38 ± 0.03	7.67 ± 0.10	7.97 ± 0.15	8.17 ± 0.15
*P. putida* S16	7.13 ± 0.06	7.43 ± 0.12	7.67 ± 0.06	7.95 ± 0.05

The experiments were carried out in the 50-mL centrifuge tube containing 10 mL of 0.9% NaCl and magnetically immobilized cells at 30 °C, 180 rpm. NaHS was added to 2 mM to initiate the reaction in each cycle and the pH was tested at the beginning and the end of each cycle

### The sulfide oxidation activities of immobilized cells are increased after repeated use and culturing

**Fig. 7:**
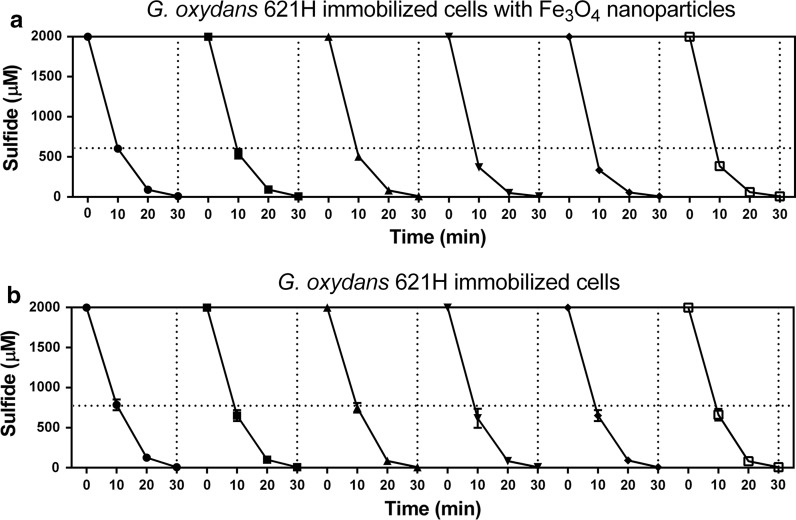
The sulfide oxidation activity of immobilized cells was increased after repeated use and culturing. The experiments were carried out in 50-mL centrifuge tube containing 10 mL of MM at 30 °C, 180 rpm. NaHS was added to 2 mM to initiate the reaction in each cycle and was completely oxidized within 40 min. After each reaction, the immobilized cells were washed with water and re-incubated in fresh MM with glucose. The cycle of sulfide oxidation was performed every 12 h. **a** *G. oxydans* 621H immobilized cells with Fe_3_O_4_ nanoparticles; **b** *G. oxydans* 621H immobilized cells. Averages (n ≥ 3) with standard deviations (error bar) were shown

**Fig. 8:**
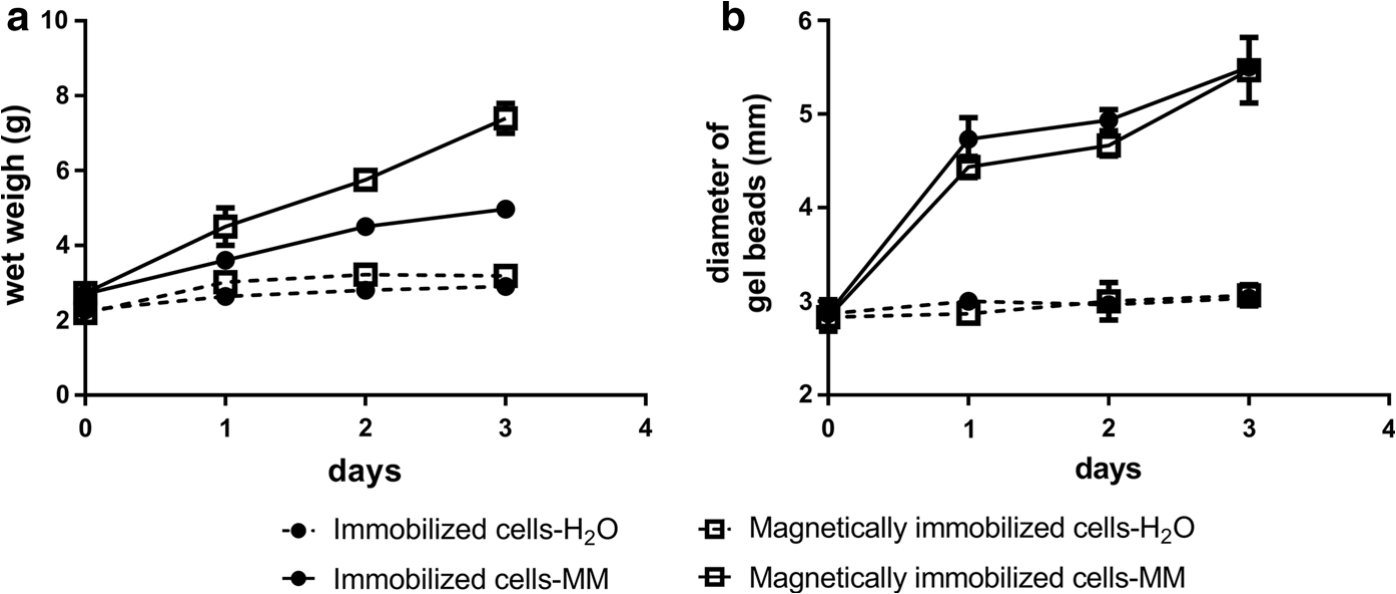
The change of wet weigh and diameter of the gel beads with immobilied *G. oxydans* 621H cells during culturing in distilled waster or MM. The cells were immobilized in alginate gel beads with or without Fe_3_O_4_ nanoparticles. The experiments were carried out in 50-mL centrifuge tubes containing 10 mL of MM or distilled water at 30 °C, 180 rpm. **a** The change of wet weigh of immobilized-cell beads. **b** The change of diameters of immobilized-cell beads. Averages (n ≥ 3) with standard deviations (error bar) were shown

**Fig. 9:**
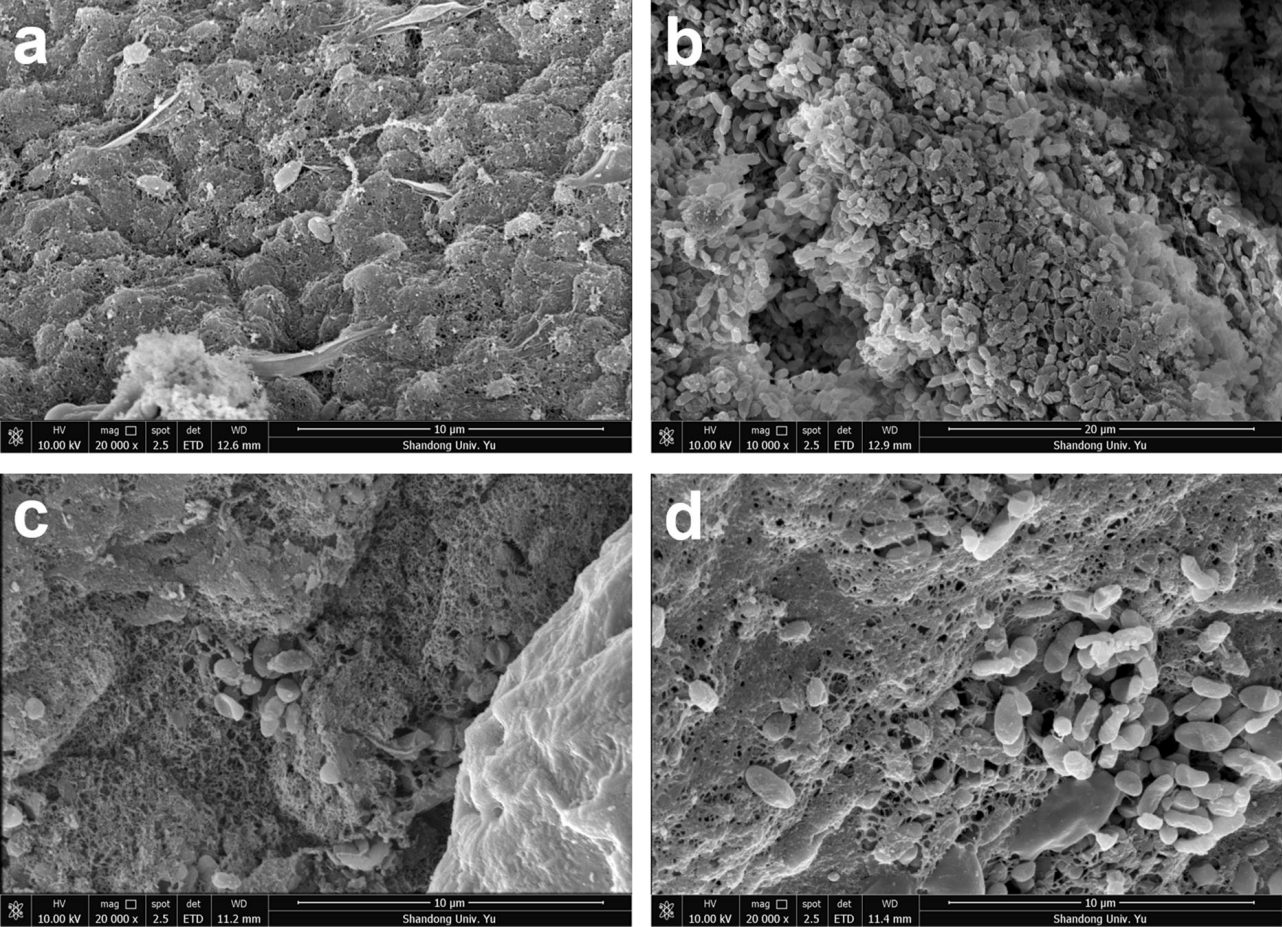
SEM images of immobilized *G. oxydans* 621H cells. The cells were immobilized in alginate gel beads or alginate gel beads with Fe_3_O_4_ nanoparticles. After 3 days of sulfide oxidation and incubation (Fig. 7 legend), the beads were analzyed by SEM. The *G. oxydans* 621H cells were embedded in the gel beads. **a** Inside of the gel beads; **b** inside of the gel beads with Fe_3_O_4_ nanoparticles; **c** surface of the gel beads; **d** surface of the gel beads with Fe_3_O_4_ nanoparticle

The activities of immobilized cells with or without Fe_3_O_4_ nanoparticles were repeatedly tested six times in 3 days, and the beads were culture in MM with 0.5% of glucose between testing. As shown in Fig. 7a, the sulfide oxidation activities of immobolized cells with Fe_3_O_4_ nanoparticles were obviously increased after 6 days’ use; they oxidized 2000 μM sulfide to about 600 μM in 10 min during the first cycle. After 3 days of sulfide-oxidation and culturing in MM, they oxidized 2000 μM sulfide to less than 400 μM in 10 min (Fig. 7a). Further, the sulfide oxidation activities of immobilized cells without Fe_3_O_4_ nanoparticles also increased to a less degree after three days (Fig. 7b). We then measured the wet weigh and diameter of gel beads over the 3 days. The diameters of the immobilized-cell beads with or without Fe_3_O_4_ nanoparticles increased to the same degree (Figs. 8b, S4), but the wet weigh of the beads with Fe_3_O_4_ nanoparticles increased more than the beads without (Fig. 8a). The increase was due to cell growth because the beads incubated in distilled water did not increase in both size or weight (Fig. 8). The results suggests that the gel beads with Fe_3_O_4_ nanoparticles has greater biomass carrying capacity. This possibility was further investigated by using scanning electron microscope (SEM) images of *G. oxydans* 621H immobilized in alginate gel beads (Fig. 9). After 3 days’ culturing, *G. oxydans* 621H had more biomass in gel beads with Fe_3_O_4_ nanoparticles than that in gel beads without Fe_3_O_4_ nanoparticles, both inside (Fig. 9a, b) and on the surface (Fig. 9c, d). More loose pores were observed in gel beads with Fe_3_O_4_ nanoparticles (Fig. 9), which may facilitate nutrient transfer and provide space for cells to grow.

## Discussion

The common presence of sulfide-oxidizing activities in heterotrophic bacteria has only recently been recognized (Luebke et al. 2014; Xia et al. 2017). In this report, some of them are shown to be effective for sulfide oxidation (Fig. 1), and the finding may promote their use in H_2_S biotreatment. Heterotrophs rely on organic compounds as the source of both carbon and energy source for growth, and they are likely oxidize H_2_S for detoxification (Luebke et al. 2014; Xia et al. 2017). Sulfur-oxidizing chemolithotrophs oxidize sulfide to gain energy for growth; and anaerobic photoautotrophic bacteria use H_2_S to provide the reducing power for photosynthesis (Syed et al. 2006); they are commonly applied in the biological treatment of H_2_S under aerobic and anaerobic conditions (Ferrera et al. 2004; Janssen et al. 1997; Krayzelova et al. 2015). The finding that some heterotrophic bacteria can rapidly oxidize sulfide offers additional choices for H_2_S biotreatment.

As previously reported, the photoautotrophic *Chlorobium* has a sulfide removal rate of 12 µmol min^−1^ g^−1^ of cell dry weight (Kim and Chang 1991). The maximum sulfide oxidation rate of *Thiobacillus denitrificans* is reported as high as 80 µmol min^−1^ g^−1^ of cell dry weight under anaerobic conditions, using nitrate as the electron acceptor (Sublette and Sylvester 1987). *Acidithiobacillus* spp. can tolerate acidic condition, and they have also been used for H_2_S biotreatment, although their preferred substrates are metal sulfides or elemental sulfur (Ben Jaber et al. 2016b). Under aerobic and acidic conditions, *Acidithiobacillus thioparus* TK-m oxidizes H_2_S at a rate higher than 59 µmol min^−1^ g^−1^ of cell dry weight, which is estimated based on the H_2_S load rate for complete removal (Kanagawa and Mikami 1989). *Thiobacillus thiooxidans* and *T. ferrooxidans* show sulfide removal rates of about 158 and 48 µmol min^−1^ g^−1^ of cell dry weight, respectively, under acidic conditions (Oprime et al. 2001). *Gluconobacter oxydans* 621H showed rate of sulfide removal closely behind those of chemolithotrophs, and *Pseudomonas* spp. also had adequate rates for sulfide oxidaiton (Table 1). Thus, heterotrophic bacteria have the potential in sulfide biotreatment, especially when they can grow fast on consuming organic compounds and co-oxidize H_2_S. They can also be cultured, harvested, and used an agent for H_2_S treatment in reactor or in the field.

Immobilized cells can effectively avoid the loss of biomass and possess better stability compared to the suspended cells in sulfide removal reactor (Kim et al. 2008). Fe_3_O_4_ nanoparticles can be economically produced, and they can be directly used for oxidizing sulfide in sewers (Lin et al. 2017). Further, Fe_3_O_4_ nanoparticles are widely used as additives for bacterial immobilization because the nanoparticles can reduce the mass transfer resistance inside the gel beads and facilitate bioremediation of organic pollutants and removal of heavy metals (Wang et al. 2007; Zhang 2003). We demonstrate that the addition of Fe_3_O_4_ nanoparticles into gel beads produces more space inside the beads, facilitating nutrient transfer, providing space for growth, and resulting in an increased sulfide oxidation activity in repeated use and culturing (Figs. 7, 8, 9). The Fe_3_O_4_ nanoparticles also catalyzed chemical oxidation of sulfide, possibly due to the presence of Fe^3+^ in Fe_3_O_4_ (Li et al. 2008). On the basis of 30% sulfide oxidation by Fe^3+^, theoretically only 7% of Fe_3_O_4_ was consumed after six oxidaton cycles of total 12 mM sulfide (Fig. 7).

Most significantly, we showed *G. oxydans* 621H and *P. putida* S16 immobilized cells oxidize sulfide without causing acidification, probably due to the production of thiosulfate and zero-valence sulfur instead of sulfuric acid. The oxidation of H_2_S to zero-valence sulfur consumes protons, which could be the reason for the slight increase of pH. In our test, O_2_ was not a limiting factor, especially for *G. oxydans* 621H that mainly produced zero-valence sulfur. Even when it was initially consumed before the first sampling time point, it was reintroduced during sampling. In comparison, a strict control of O_2_ levels is required to minimize sulfuric acid production by sulfur-oxidizing chemotrophic bacteria (Pokorna and Zabranska 2015).

The production of thiosulfate and zero-valence sulfur is in agreement with our previous report that recombinant *E. coli* with SQR and POD oxidize sulfide to sulfite and thiosulfate (Xin et al. 2016). Interestingly, these wild type bacteria with SQR and PDO did not accumulate sulfite. This is likely due the slow polysulfide oxidation in these bacteria, and the produced sulfite rapidly reacts with the accumulated polysulfide to produce thiosulfate (Xin et al. 2016). We also detected tetrathionate as a major product after sulfide oxidation by *P. putida* S16 and *P. aeruginosa* PAO1, which is likely due to the presence of TsdA in the bacteria. Thus, the production of zero-valence sulfur, thiosulfate, and tetrathionate may prevent acidification after immediate sulfide oxidation. However, thiosulfate and tetrathionate may be further oxidized by microorganisms (Lenk et al. 2012), and the oxidation may be minimized by eliminating or inhibiting bacteria with TsdA.

H_2_S emission is a problem in sewer systems mainly due to its corrosion of concrete pipes. Traditional chemical methods of remediation bring huge costs to urban governance (Zhang et al. 2008). Considering sulfide oxidation by heterotrophic bacteria does not cause apparent acidification and the magnetically immobilized heterotrophs with Fe_3_O_4_ nanoparticles are conveniently recyclable, our finding may provide a new way to control the H_2_S corrosion in sewer systems. Further, many heterotrophic bacteria have the ability to degrade organic pollutants, such as pesticides, and to immobilize heavy metals (Cycoń et al. 2017; Mulligan 2005). Therefore, heterotrophic bacteria may potentially degrade organic pollutants as well as removing sulfide.

In conclusion, we identified that heterotrophic bacteria with *sqr* and *pdo* had the ability to oxidize exogenous sulfides and some of them have comparable oxidation rates with those of chemolithotrophic bacteria. In addition, the fast growth and no-acidification offer some advantages for H_2_S removal. Since these heterotrophic bacteria with *sqr* and *pdo* are abundant and diverse in nature (Xia et al. 2017), many of them may potentially be used for sulfide biotreatment. Thus, this report may promote the use of heterotrophic bacteria in H_2_S biotreatment. At least, they can be used as alternative choices rather than chemolithotrophs for H_2_S bioremediation. Further, since they are common in nature (Xia et al. 2017), they may be simply used for in situ H_2_S oxidation.

## Electronic supplementary material

Below is the link to the electronic supplementary material.

Supplementary material 1 (PDF 2764 kb)
