# Supplementary material for: H2S biotreatment with sulfide-oxidizing heterotrophic bacteria
Source: Biodegradation. 2018 Aug 23;29(6):511–24. doi: 10.1007/s10532-018-9849-6 (PMC6245092; doi:10.1007/s10532-018-9849-6)
Supplement: Supplementary file 1 — Supplementary material 1 (PDF 2764 kb) [file 10532_2018_9849_MOESM1_ESM.pdf]

Supplementary Material for *Biodegradation*

H_2S biotreatment with Heterotrophic Sulfide-Oxidizing Bacteria

Ningke Hou[1], Yongzhen Xia[1], Xia Wang[1], Huaiwei Liu[1], Honglei Liu[1,]*, and Luying Xun[1,2],*

[1] *State Key Laboratory of Microbial Technology, Shandong University, Jinan, 250100, P. R. China.*

[2] *School of Molecular Biosciences, Washington State University, Pullman, WA 99164-7520, USA.*

***For correspondence:**

Honglei Liu: Email lhl@sdu.edu.cn; Tel. + 86 159-6664-2788

Luying Xun: Email luying_xun@vetmed.wsu.edu; Tel. +1 509-335-2787; Fax +1 (509) 335 4159.

Contents:

Figure S1. The heat-inactive cells in FIG 1b did not oxidize sulfide.

Figure S2. The end-products of sulfide oxidation by five bacteria after 6-hours reaction

Figure S3. Magnetic beads without cells oxidize sulfide

Table S1. Tested heterotrophic bacteria and SQR and PDO accession numbers

Table S2. Distribution of TsdA in different bacterial phyla

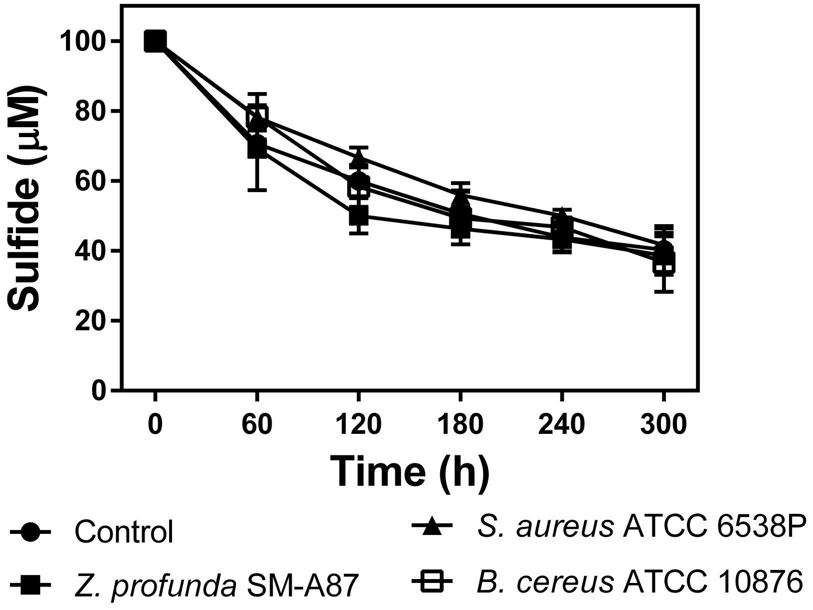

Fig. S1. The heat-inactive cells in FIG 1b did not oxidize sulfide. Cells were cultured, harvested, wash and re-suspended in the same way with FIG. 1. They were then heated in boiling water 30 minutes. NaHS was added to 100 μM to initiate the reaction. Averages (n⩾3) with standard deviations (error bar) were shown.

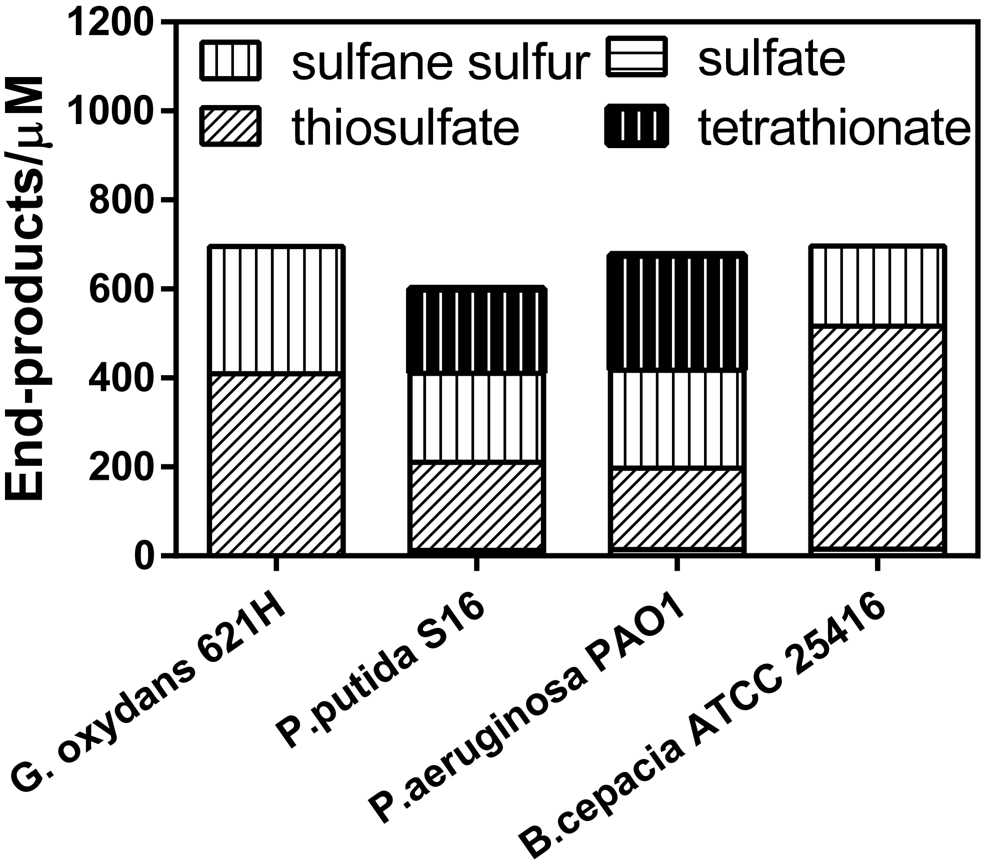

Fig. S2. The end-products of sulfide oxidation by five bacteria after 6-hours reaction. The experiments were done as described in the Figure 1 legend. Thiosulfate contains two sulfur atoms and tetrathionate contains four sulfur atoms, theirs' concentration was adjusted. Sulfane sulfur, zero valence sulfur.

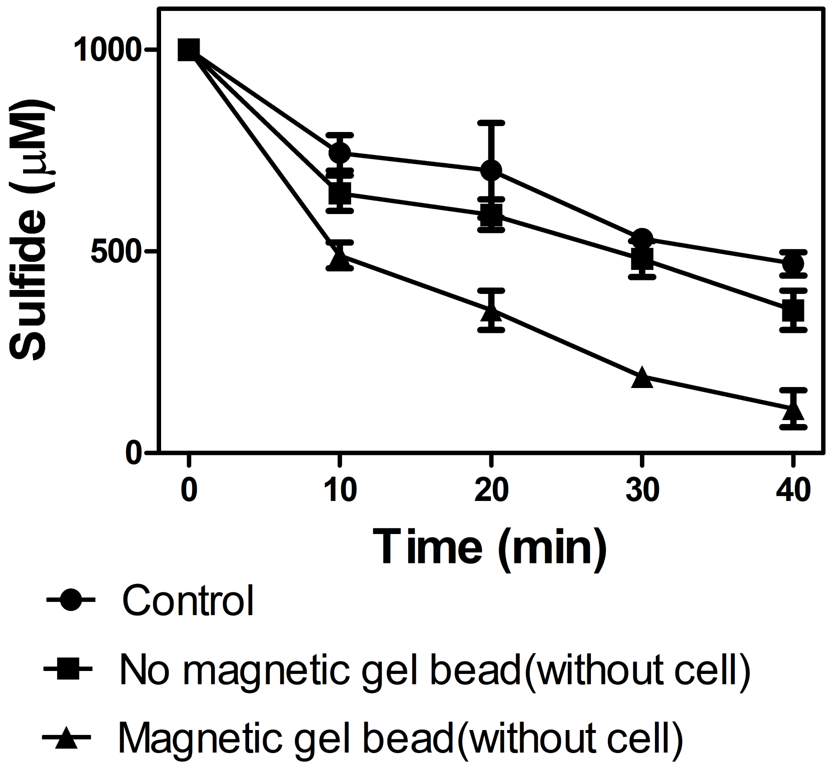

Fig. S3. Gel beads coated Fe_3O_4 nanoparticles without cells oxidize sulfide. The experiments were carried out in 50-ml centrifuge tube containing 10 ml of HEPES buffer (pH=7.4) at 30°C on a reciprocal shaker at 180 r.p.m.. Averages (n≥3) with standard deviations (error bar) were shown.

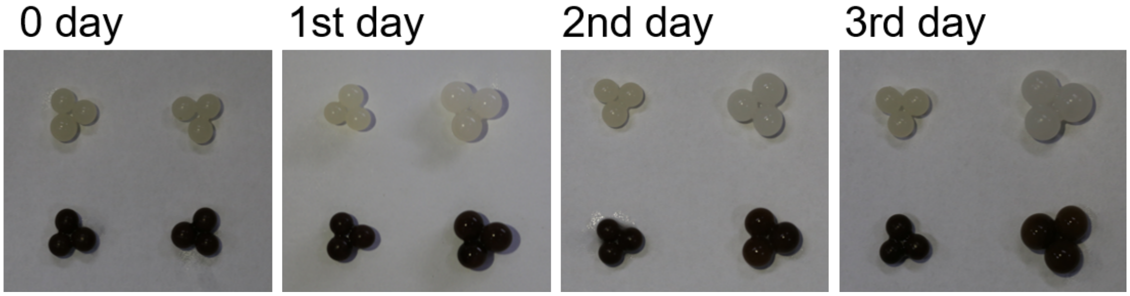

Fig. S4. The change of diameters of beads with immobilized *G. oxydans* 621H cells during culturing in water or MM. The cells were immobilized in alginate gel beads with (black) or without (white) Fe_3O_4 nanoparticles. The experiments were carried out in 50-ml centrifuge tube containing 10 ml of distilled water or MM at 30°C on a reciprocal shaker at 180 r.p.m. On the left is the immobilized-cell beads incubated in distilled water; on the right is the immobilized-cell beads incubated in MM with glucose.

Table S1. Tested heterotrophic bacteria and SQR and PDO accession numbers

Strains	SQR accession No.	PDO accession No.
G. oxydans 621H	WP_011252149.1<br>WP_011252513.1	WP_011252150.1
P. putida S16	WP_013970253.1	WP_013970252.1
P. aeruginosa PAO1	NP_251256.1<br>NP_251035.1	NP_251605.1
B. cepacia ATCC 25416	WP_027787303.1	WP_027790374.1<br>WP_027787304.1
B. cereus ATCC10876	WP_000857315.1	WP_000925319.1
Z. profunda SM-A87	WP_009779527.1	WP_009779529.1
S. aureus ATCC 6538P	WP_001033018.1	WP_000465474.1
S. marcescens ATCC13880	WP_033641274.1<br>WP_033640364.1	WP_033640361.1
E. coli BL21	None	None

Table S2. Distribution of TsdA in different bacterial phyla.

	TsdA couts
Betaproteobacteria	553
Gammaproteobacteria	294
Alphaproteobacteria	115
Epsilonproteobacteria	91
Bacilli	11
Synechococcales	9
Flavobacteriia	8
Sphingobacteriia	7
Deltaproteobacteria	6
Nitrospirales	4
Acidobacteriales	3
Parachlamydiales	2
Aquificales	2
Chitinophagia	2
Deinococci	2
Gemmatimonadales	2
Cytophagia	1
